# Responses of Free-Living Planktonic Bacterial Communities to Experimental Acidification and Warming

**DOI:** 10.3390/microorganisms11020273

**Published:** 2023-01-20

**Authors:** Anastasia Tsiola, Evangelia Krasakopoulou, Daniele Daffonchio, Constantin Frangoulis, Tatiana M. Tsagaraki, Stilianos Fodelianakis, Paraskevi Pitta

**Affiliations:** 1 Institute of Oceanography, Hellenic Centre for Marine Research, 71003 Heraklion Crete, Greece; 2 Institute of Oceanography, Hellenic Centre for Marine Research, 19013 Anavyssos Attiki, Greece; 3 Red Sea Research Center, Biological and Environmental Sciences and Engineering Division (BESE), King Abdullah University of Science and Technology (KAUST), Thuwal 23955-6900, Saudi Arabia; 4 Department of Biological Sciences, University of Bergen, 5006 Bergen, Norway

**Keywords:** climate change, small bacteria, acidification, warming, mesocosms

## Abstract

Climate change driven by human activities encompasses the increase in atmospheric CO_2_ concentration and sea-surface temperature. Little is known regarding the synergistic effects of these phenomena on bacterial communities in oligotrophic marine ecosystems that are expected to be particularly vulnerable. Here, we studied bacterial community composition changes based on 16S rRNA sequencing at two fractions (0.1–0.2 and >0.2 μm) during a 10- day fully factorial mesocosm experiment in the eastern Mediterranean where the pH decreased by ~0.3 units and temperature increased by ~3 °C to project possible future changes in surface waters. The bacterial community experienced significant taxonomic differences driven by the combined effect of time and treatment; a community shift one day after the manipulations was noticed, followed by a similar state between all mesocosms at the third day, and mild shifts later on, which were remarkable mainly under sole acidification. The abundance of *Synechococcus* increased in response to warming, while the *SAR11* clade immediately benefited from the combined acidification and warming. The effect of the acidification itself had a more persistent impact on community composition. This study highlights the importance of studying climate change consequences on ecosystem functioning both separately and simultaneously, considering the ambient environmental parameters.

## 1. Introduction

Anthropogenic perturbations in the environment are rarely one-dimensional [1]. In fact, climate change is caused by anthropogenic activities and includes changes in atmospheric, terrestrial and aquatic ecosystems, i.e., increased water levels, increased concentrations of atmospheric CO_2_ and loss of ice in the Northern Hemisphere [2]. Although the oceans act as “buffers” upon the intrusion of CO_2_ in surface waters, an inevitable decrease in the pH (i.e., ocean acidification effect) has already been detected, averaging approximately 0.1 units since the beginning of the industrial era [3]. Warming of the surface oceans is the other alteration that occurs concomitantly with acidification, and it is suspected that until 2100, surface-water temperatures may increase up to 6 °C [4].

The warming of the oceans began to be studied earlier compared to acidification. Plenty of findings indicate that phytoplankton with a small cell size (i.e., in a range between pico- and nano- plankton) is favored [5,6] and phytoplankton biomass is increased upon experimental warming [7]. On the other hand, experimental acidification has been found to benefit the productivity of large phytoplankton cells [8,9] and release of organic matter [10] but also to suppress the growth of smaller cells, with non–homogeneous responses over time [11,12,13]. Noteworthy, a recent review of ocean–acidification mesocosm experiments highlighted significant impacts even on the C/N ratios of the organic material exported from the water column [14].

Regarding the impacts of ocean acidification on heterotrophic bacterial extracellular enzymatic activity, variable responses have been documented so far and in most cases, they were attributed to changes in the phytoplankton community [15,16,17]. Indeed, the organic matter released by phytoplankton was primarily affected by acidification and subsequently it was also found to influence bacterial enzymatic activities and viral production rates [18]. Still, variability in the currently–available reports exists, since the environmental and experimental conditions vary too [19]. Among the variable environmental terms, the original water temperature, light intensity and nutrient levels, or in other words, the ecosystem trophic status, seem to determine the functional vs. morphological vs. taxonomic microbial composition changes upon pH [20,21,22] and temperature manipulation [23]. Most importantly, notwithstanding the challenges of extending the outcomes of the above–mentioned short–term studies to real ocean acidification effects, most of them have done so [24].

Few studies have focused on the simultaneous impacts of ocean warming and acidification to non–calcifying organisms. So far, a combination of the two phenomena has been found to enhance *Synechococcus* [25,26] and inhibit *Prochlorococcus* growth [25], alter bacterial community composition [27] and increase phytoplankton biomass [28] and, especially the biomass of small phytoplankton [29]. Synergistic effects have been largely overlooked in the oligotrophic seas, although it is known that the potential synergies would be more drastic in nutrient–limited regions; in fact, a more–acidic future ocean is assumed to be more vulnerable to secondary chemical and physical perturbations. Additionally, ambient temperature seems to determine the impacts of the pH reduction [30], ultimately making the simultaneous study of these factors a vital methodological aspect.

The eastern Mediterranean Sea (EMS) is one of the most oligotrophic regions at the global scale [31], where the thermal stratification and nutrient depletion may affect ecosystem responses to acidification and warming stronger than other regions [32,33,34]. The EMS has a naturally-high capacity to absorb but also buffer anthropogenic CO_2_, as its waters have a high total alkalinity, being on average 10% higher than the global ocean, as well as a unique overturning circulation [35]. The acidification rate of EMS surface waters has been estimated to be at the 0.08 pH_T_ unit (total hydrogen ion scale) since 1800, which is comparable to that of the global ocean, despite having a 10% greater anthropogenic carbon inventory [35]. For the EMS, the decrease in pH compared to the pre-industrial era ranges between 0.242 and 0.457 pH units according to the most optimistic (B1) and pessimistic (A1F) of the IPCC (2007) scenarios, respectively [36]. However, considering the concomitant increase in seawater temperature, the penetration of atmospheric CO_2_ into the seawater would decrease, the penetration of anthropogenic carbon in seawater would slow down and the range of the pH decrease in 2100 would be lower, between 0.19 and 0.37 pH units [36].

Recognizing that the currently–published work originates from various ecosystems rather than the oligotrophic EMS, the novel work presented here was designed in order to fill the gap concerning future climate change scenarios under extreme oligotrophy. Indeed, microbial-mediated processes within the oceans are expected to be of critical importance in view of climate-related alterations such as increased stratification [37]. Recently, Cavicchioli et al. [38] raised concerns regarding the fate of the oceans if marine microbes and their interactions are not considered and studied properly. Our contribution in this direction is hereby presented; bacterial community composition was monitored over a 10–day mesocosm experiment in the Cretan Sea, EMS, where the pCO_2_ and temperature were modified to mimic future oceanic scenarios of climate change (pH decreased by ~0.3 units; temperature increased by ~3 °C). Mesocosm experiments are now seen as an irreplaceable experimental tool to explore aquatic food web responses to and interactions with factors such as ocean acidification and warming. A fully factorial mesocosm experiment was originally designed to study the individual and concomitant effects of the treatments in microbial plankton: including the abundance of virus-like particles, autotrophic and heterotrophic bacteria, pico-, nano- and micro- sized eukaryotic cells, concentration of chlorophyll, production rates of heterotrophic bacteria and primary producers, phosphorus and nitrogen uptake rates, and community responses of coccolithophores (the latter presented in D’ Amario et al. [39]) as well as the associated chemical parameters: including dissolved and particulate inorganic and organic nutrient concentrations, and carbonate-system related parameters, in triplicate mesocosms. Our special focus in this manuscript was on bacterial community diversity based on PCR amplification of the 16S rRNA gene at two size fractions (0.1–0.2 and >0.2 μm), which is presented for selected experimental days (before warming/acidification amendments, and then after 1, 3, 6 and 10 days). The taxonomic differences were tested for statistically significant differences and for correlation with the treatments (i.e., pH and temperature values) and the most relevant abiotic variables (i.e., chlorophyll a, inorganic nitrogen and phosphate concentrations).

## 2. Materials and Methods

### 2.1. Experimental Setup and Sampling

Seawater was collected from a site ~5 nautical miles north of Heraklion port in the Cretan Sea, Mediterranean Sea (degree of latitude and longitude: 35° 24.957 N, 25° 14.441 E) and transported within two hours to the CretaCosmos mesocosm facility (https://www.aquacosm.eu/mesocosm/cretacosmos/) of the Hellenic Centre for Marine Research (HCMR). Subsurface water (10 m) was retrieved on board the R/V *Philia* using a submersible pump. Upon return to the mesocosm facility, the water was distributed evenly through gravity siphoning with acid cleaned and deionized water rinsed plastic tubes into 12 low-density polyethylene bags to a final volume of 3 m^3^ each. The collection of seawater was conducted on the 30 and 31 August 2013, and it was transported to the facility using 1 m^3^ high-density polyethylene (HDPE) barrels that had been washed with acid (10% HCl) and rinsed with deionized water three times prior to filling.

The mesocosm bags were deployed in two large concrete tanks (150 m^3^ and 350 m^3^ water volume capacities). Six of them were incubated in the first tank at *in situ* temperature that was kept constant with a continuous water–flow system, while the remaining six bags were incubated in the second tank at +3 °C compared to the *in situ* temperature. The design of the tank arrays, according to Comwal and Hurdl [19], was A-3 (i.e., systematic, having a certain degree of randomness). We used a Plexiglas lid to protect the mesocosms from atmospheric deposition and a mesh screen to mimic the light conditions at the sampling depth (10 m). The initial abiotic and biotic conditions of the seawater were determined by sampling the mesocosms at T–1 (1 September 2013). Altogether, 3 mesocosms were labeled after “Warming” (W: only warming), 3 after “Ocean Acidification” (OA: only addition of CO_2_) and 3 others after “GreenHouse” (GH: warming and addition of CO_2_). The last 3 mesocosms were not treated and served as the control (C). All treatments were performed in triplicates (1, 2 and 3). After the T–1 sampling, three mesocosms from each tank were treated with CO_2_-saturated seawater in order to achieve final pCO_2_ levels equivalent to the expected pH decrease in the Eastern Mediterranean by the end of the century under the most pessimistic IPCC SRES scenario, i.e., the A1F1 (IPCC, 2007). The details of the procedure are presented here and in D’ Amario et al. [39]: part of the seawater that was collected for the filling of the mesocosm bags was stored in two large–volume (100 L) dark plastic containers, kept away from direct sunlight exposure and was actively bubbled for several minutes with pure CO_2_ to achieve saturation. Then, the saturated-in-CO_2_ seawater was transferred in 10–L plastic Nalgene containers and added through gravity siphoning to the 6 mesocosms. Between 28.5 and 31.0 L were added, depending on the temperature of the “host” tank and the volume of each mesocosm bag. A specially–designed diffusing system [40] was used, ensuring the even distribution of the CO_2_-saturated seawater in the seawater volume in each enclosure. To minimize the stress induced by the addition of relatively-large quantities of acid water, the acidification treatment of the mesocosms was performed over 3 days. On 3 September, the ”acidification phase” was completed. No further manipulations of the CO_2_ system were performed during the experiment and pCO_2_ levels evolved in the mesocosms mainly due to biological activity and, to a lesser degree, due to the air/sea exchanges of CO_2_.

Samples for the bacterial community composition assessment were collected at T–1 (1 September 2013), T0 (2 September 2013), T3 (5 September 2013), T6 (8 September 2013) and T10 (12 September 2013) from two replicates of each treatment (C1-2, W1-2, OA1-2 and GH1-2). For this analysis, approximately 10 L of seawater from these mesocosms were placed in acid-cleaned and deionized-water-rinsed, low-density polyethylene containers and filtered firstly through a 3 μm polycarbonate filter to remove large particles and then through 0.2 μm polycarbonate filters for bacterial cells using a vacuum pump (Millipore, MA, USA) performing at a low vacuum pressure (<150 mmHg) and a 47–mm diameter filtration apparatus (Thermo Scientific™ Nalgene™ Reusable Filter Holders with Receiver). This fraction represents the free-living >0.2 μm bacterial community. Then, the 0.2–μm filtrate was collected in a new container (also acid-cleaned and deionized-water-rinsed) and filtered again through 0.1 μm polyethersulfone filters for bacterial cells <0.2 μm, using the same vacuum pump and a 50-mm in-line filter holder apparatus (Sartorius, Göttingen, Germany). This fraction represents the free-living 0.1–0.2 μm bacterial community. Both 0.2 and 0.1 μm filters were flash-frozen in liquid nitrogen and stored at –80 °C pending DNA extraction.

### 2.2. pCO_2_ and Temperature Determination

The sampling for the pH and total alkalinity determination was performed in all mesocosms every day starting at T–1, separately from the other parameters with the recommended precautions [41] and as presented in D’ Amario et al. [39]. The samples were analyzed within few hours after collection. The pCO_2_ was calculated with the R package *seacarb* [42], using pH_T_ and A_T_ as well as temperature and salinity based on the CTD measurements performed daily. Seawater pH on the total scale (pH_T_) was measured with a pH meter (Metrohm, 827 pH lab) fitted with a glass electrode (Metrohm, Aquatrode Plus) calibrated on the total hydrogen ion concentration scale using Tris/HCl buffer solution [41] with a salinity of 38.0 provided by A. Borges (University of Liege, Belgium). The standard deviation of the pH of the Tris/HCl buffer measured at 25.4 °C during the whole experiment was 0.01. The total alkalinity was measured with potentiometric titration using a VINDTA 3S analyzer (Versatile INstrument for the Determination of Total inorganic carbon and titration Alkalinity) in duplicate samples directly preserved after sampling by adding saturated mercuric chloride (HgCl_2_) aqueous solution at 0.02% by sample volume. The accuracy and reproducibility were checked using Certified Reference Material (CRMs, Batch 82) supplied by A. Dickson (Scripps Institution of Oceanography, USA); measured A_T_ value 2335.8 ± 1.8 μmol kg^−1^, *n* = 12, certified value: 2335.02 ± 0.25 μmol kg^−1^. The pCO_2_ uncertainty in the different sets of mesocosms estimated with the “errors” function of *seacarb* and based on the standard deviations of pH_T_ and A_T_ [43]. Thus, the combined uncertainty for the pCO_2_ ranged between 12.0 and 21.2 μatm, being lower in the C and W mesocosms and higher in the OA and GH ones.

From an initial range of pCO_2_ levels between 362 and 371 μatm (corresponding pH range; 8.10–8.11, T–1, *n* = 8), the pCO_2_ increased to 549 and 533 μatm at OA1 and OA2 (corresponding pH: 7.97 and 7.98) and to 612 and 611 μatm at GH1 and GH2 (corresponding pH: 7.93 at both cases) at T0 (Table 1). At T2, the “acidification phase” was completed. At T3, the pCO_2_ was 794 and 768 μatm at OA1 and OA2 (corresponding pH: 7.84 and 7.85) and 803 and 872 at GH1 and GH2 (corresponding pH: 7.83 and 7.80) (Table 1)

The temperature was measured using Aanderaa CT sensor 3919 sensors (Aanderaa Data Instruments) and HOBO UA-002-64T sensors (Onset Computer Corporation, Bourne, USA). From an initial average level of 26.08 ± 1.04 °C (T–1, *n* = 8), the temperature increased to 27.46 and 27.40 °C at W1 and W2, and to 27.35 and 27.34 °C at GH1 and GH2 at T0, respectively (Table 1). During the whole experimental period, the tank water temperature remained at 25.0 ± 0.3 °C in the lower-temperature tank and 27.7 ± 0.5 °C in the higher-temperature tank. The temperature difference between mesocosms placed in the same tank was always <0.4 °C (at 80% cases, it was <0.2 °C).

### 2.3. DNA Extraction, Library Preparation, Sequencing and Sequence Processing

**Figure 1:**
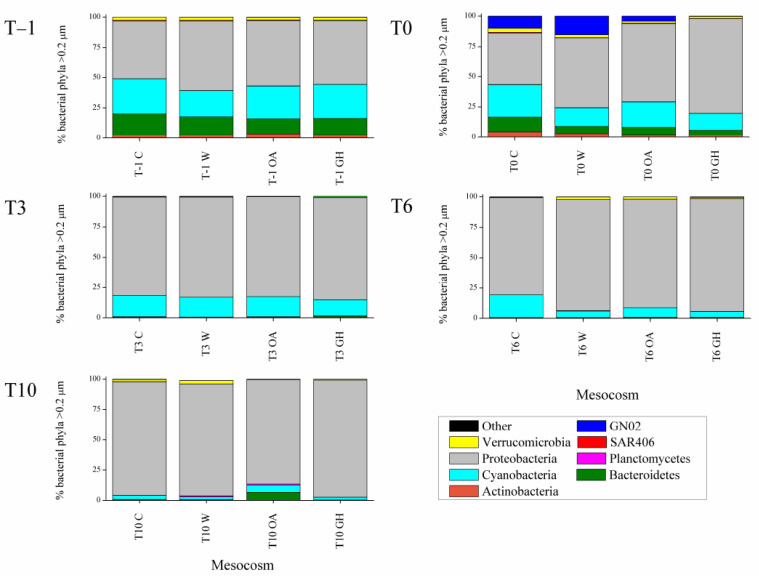
Percentage contribution of bacterial phyla (>0.2 μm fraction) in the mesocosms at T–1, T0, T3, T6 and T10. “Other” phyla include Acidobacteria, Chlorobi, Chloroflexi, Firmicutes, Gemmatimonadetes, NC10, Nitrospirae, Spirochaetes and ZB3. C: control mesocosms, W: mesocosms where the temperature was increased by 3 °C (labeled after warming), OA: mesocosms where the pH was decreased by 0.3 units (labeled after ocean acidification), GH: mesocosms where the temperature was increased by 3 °C and the pH was decreased by 0.3 units (labeled after greenhouse).

**Figure 2:**
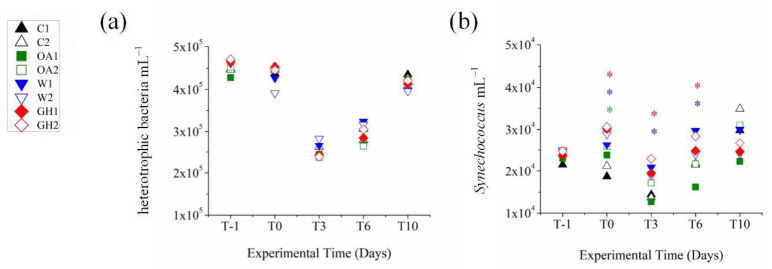
The abundances of (**a**) heterotrophic bacteria (cells mL^−1^) and (**b**) *Synechococcus* (*Synechococcus*, cells mL^−1^) in the mesocosms at T–1, T0, T3, T6 and T10. Colored asterisks above symbols denote significant differences from the controls. For the treatment abbreviations, please refer to Figure 1.

**Figure 4:**
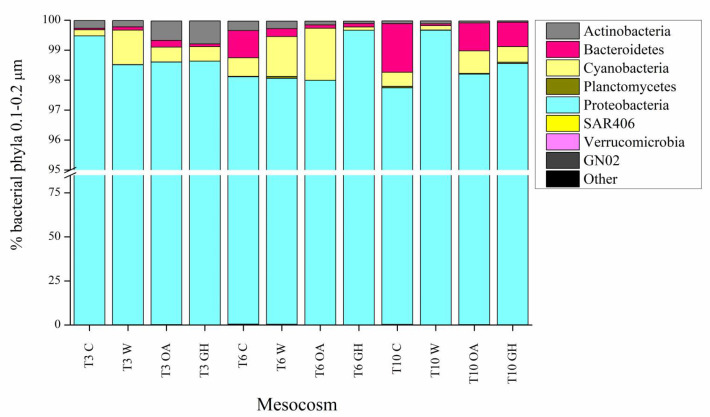
Percentage contribution of bacterial phyla (0.1–0.2 μm fraction) in the mesocosms at T3, T6 and T10. “Other” phyla include Acidobacteria, Chlamydiae, Chloroflexi, Firmicutes, TM6, TM7 and ZB3. For the treatment abbreviations, please refer to Figure 1.

The DNA was extracted from 0.2 and 0.1 μm filters following the CTAB protocol of Winnepenninckx [44] as described elsewhere [45]. Specifically, the frozen 0.1 and 0.2–μm filters were ground with a mortar and pestle in a continuous flow of liquid nitrogen. The ground filters were incubated at 60 °C for 2 h at ~2 turns min^−1^, with 10 mL of 2% CTAB buffer. The DNA was then purified by centrifugation (7500 rpm, 10 min, 4 °C) using an equal volume of chloroform:isoamylalcohol solution. The aqueous phase was treated with RNase and the chloroform:isoamylalcohol step was repeated. The DNA precipitation was performed overnight by adding isopropanol (two/thirds of the volume of the aqueous phase), followed by centrifugation (7500 rpm, 15 min, 4 °C). The DNA pellets were washed with a solution of 76% *v*/*v* ethanol and 10 mM ammonium acetate. The extracted DNA was dissolved in ultrapure water and quantified using the Qubit^®^ high–sensitivity assay kit in a 3.0 QubitTM fluorometer (Thermo Fisher Scientific^TM^, Massachusetts, USA). The DNA yields varied between ~5 and ~20 μg. PCRs were carried out (25 μL final volume) for the 16S rRNA gene using the locus-specific primers 341f (5-CCTACGGGNGGCWGCAG-3) and 805RB (5-GGACTACNVGGGTWTCTAAT-3) following Klindworth et al. [46] and Apprill et al. [47] and a universal 5′ tail specified by Illumina. The first and second PCR reaction mixture and amplification conditions and the normalization and purification steps were performed as described in Tsiola et al. [45]. The sequencing was conducted in the Illumina Miseq 4000 platform available at KAUST Bioscience Core Lab using paired-end sequencing. In total, 16S rRNA amplicons were sequenced and processed from 64 samples (from the >0.2 μm fraction: 8 mesocosms (C1, C2, W1, W2, OA1, OA2, GH1 and GH2) × 5 time points (T–1, T0, T3, T6 and T10) and from the 0.1–0.2 μm fraction: 8 mesocosms (C1, C2, W1, W2, OA1, OA2, GH1 and GH2) × 3 time points (T3, T6 and T10)). The DNA was not pooled at the laboratory, and the sequences were not pooled during the bioinformatic analysis. Since the standard deviation cannot be calculated from the duplicate values, the data in Table 1 and Figure 2 is presented for the individual mesocosm bags, while the averages of “C1-C2”, “W1-W2”, “OA1-OA2” and “GH1-GH2” are presented in 100% stacked-columns in Figure 1 and Figure 4.

Approximately 20 million raw 16S rRNA sequences were generated using Illumina MiSeq, which were then quality–checked and analyzed using both UPARSE v8 and QIIME v1.9. Paired-end reads formation, quality check in QIIME, removal of forward and reverse primers from the sequence ends of the high-quality reads and, then merging of the individual sample files was conducted as in Tsiola et al. [45]. The threshold for the high–quality reads was that each read passing the threshold contained less than 1 estimated error base, based on the read’s quality scores. The sequences after quality check and trimming were between 12,885 and 572,044 reads per sample, with an average length of 464 ± 15 bp. The single file that contained all of the sample reads was then imported in UPARSE, where operational taxonomic units (OTUs) of 97% sequence similarity were picked, and the chimeric sequences were further discarded by de–novo and reference–based detection. The “Gold” database (http://microbiomeutil.sourceforge.net/, accessed on 1 September 2016) was used for the reference–based detection and the representative sequences of the OTUs were assigned taxonomy in QIIME with UClust and searched against the newest Greengenes database. The OTU counts for each sample and the taxonomic assignments were combined into an OTU table, where the OTUs that were affiliated to Archaea and OTUs without a taxonomic assignment were not included in further analyses. The alpha- and beta-diversity analyses were performed using this resulting OTU table. The samples OA2 at T6 and T10 were removed from the dataset due to the low sequence read counts. The raw sequences have been submitted to the European Nucleotide Archive (ENA) database with accession number ERA19781743 (runs between ERR10742502 and ERR10742565).

### 2.4. Determination of the Concentrations of Nitrate, Ammonium, Phosphate and Chlorophyll a

The dissolved phosphate concentration was estimated with the MAGIC method [48]. The concentrations of dissolved nitrate were estimated according to Strickland and Parsons [49] and the concentration of dissolved ammonium according to Ivancic and Degobbis [50]. The detection limits for phosphate, nitrate and ammonium were 0.8 nM, 0.017 and 0.019 μM, respectively.

Following Yentsch and Menzel [51], a sub-sample (approx. 1 L) was filtered through 47 mm GF/F filters in order to estimate the total chlorophyll a concentration. After the gentle vacuum pumping, the filters were stored at −20 °C pending extraction in the laboratory in a 90% aceton solution (overnight). The pigment analysis was conducted using a Turner TD-700 fluorometer.

### 2.5. Determination of Bacterial Abundances

The abundance of heterotrophic and autotrophic bacteria was determined by applying flow cytometry in samples fixed with 0.2–μm filtered glutaraldehyde, based on Marie et al. [52]. Autotrophic bacteria were analyzed live without fixation and were discriminated into *Synechococcus* and *Prochlorococcus* based on the characteristic auto-fluorescence of chlorophyll and phycoerythrin. Samples for determination of heterotrophic bacterial abundance were diluted in Tris-EDTA buffer solution (pH = 8, Sigma-Aldrich) to maintain the particles’ enumeration at a rate of <1000 events sec^−1^; they were stained with SYBR Green I (Molecular Probes, Eugene, USA) at a 4 × 10^−4^ final dilution of the stock solution and then incubated for 10 min in the dark. Yellow–green latex beads of a 1 μm nominal size (Polysciences, Warrington, USA) were used as an internal standard of fluorescence. A FACSCalibur^TM^ instrument (Becton Dickinson, New Jersey, USA) was used at a conventional air pressure, with an air-cooled laser at 488 nm and a standard filter setup. The flow rate of the instrument was determined daily and used for the abundance conversion by accurately weighing a trial Tris-EDTA buffer solution sample before and after running for 5 min at all speed performances. The data was processed with the Cell Quest Pro software (Becton Dickinson).

### 2.6. Statistics

A principal coordinates analysis was applied to coordinate the data [53]. Significant differences in the bacterial patterns of the samples were tested by applying an n-factor permutational multivariate analysis of variance using the factors “time” and “treatment”. Our null hypothesis was that there were no differences. Bray-Curtis dissimilarity matrices on square-root transformed biological data were constructed [54]. Hypothesis testing was performed using 999 permutations and pairwise tests using a significance level of 0.05. The exploratory analyses were conducted with the software package PRIMER v6 (PRIMER-E Ltd., Plymouth Marine Laboratory, Natural Environmental Research Council, UK) with PERMANOVA + add-on software. A redundancy analysis (RDA) was used to summarize the variation in the biological data (bacterial genera and family percentage contribution to total genera and families) that was explained by the abiotic data. The results of this method are presented in RDA plots. Repeated-measures analysis of variance (RM ANOVA) was applied to determine the significant differences in the abundances of heterotrophic and autotrophic bacteria and the concentrations of dissolved nutrients and chlorophyll a. The significance of the differences was assessed with post-hoc Tukey tests (Tukey HSD). The homogeneity of variance was checked using Levene’s test. ANOVAs were performed using IBM SPSS Statistics software v23.

## 3. Results and Discussion

The >0.2 μm bacterial community composition significantly differed between the mesocosms at the genus level based on experimental time (PERMANOVA, Pseudo-F_4,29_ = 11.05, *p* < 0.05) and the combined effect of experimental time and treatment (PERMANOVA, Pseudo-F_12,14_ = 1.74, *p* < 0.05). At the smallest 0.1–0.2 μm fraction, the bacterial community composition varied neither with treatment nor with time.

**Figure 3:**
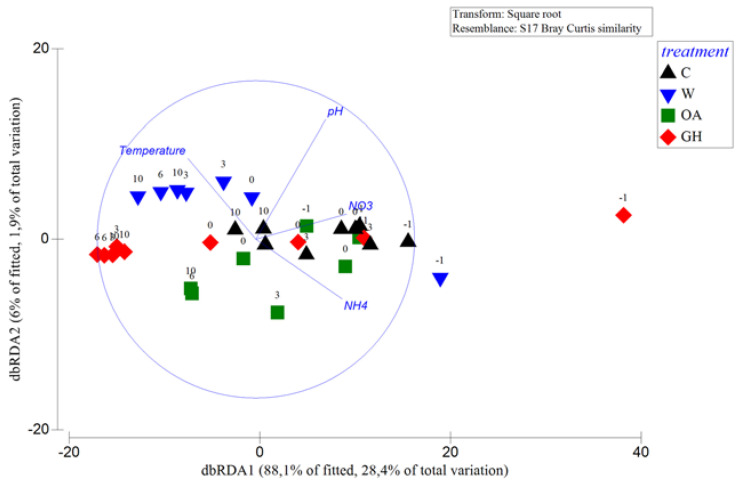
Distance-based redundancy analysis (dbRDA) plot of the distance-based linear model of the pH and temperature measurements and the concentrations of nitrate (NO_3_^-^) and ammonium (NH_4_^+^) fitted to the bacterial community composition (genus level). The length and direction of the vectors indicate their relative strength and direction of relationship in the ordination plot, respectively. Community similarity was calculated via the Bray–Curtis index after a square–root transformation of the bacterial genera read counts. The samples are presented based on the mesocosm treatment (symbols) and experimental day (numbers above symbols). For the treatment abbreviations, please refer to Figure 1.

Various taxonomic differences at the >0.2 μm bacterial fraction were seen immediately after the pH and temperature manipulation (immediate treatment effects at the >0.2 μm bacterial fraction, see 3.1. chapter below); some taxa benefited while others were inhibited or remained unaffected (Figure 1). Later during the experiment, the differences between the controls and treatments were less apparent or different compared to the initial ones (short-term treatment effects at the >0.2 and 0.1–0.2 μm bacterial fractions, see 3.2. and 3.3. chapters below), confirming that the majority of bacterial taxa may cope with the experimental pH and temperature manipulations [27,55,56,57,58]. Both pH and temperature significantly affected the bacterial community composition (distLM, adjusted R^2^ = 0.13, *p* < 0.05). We found that W and GH shared similar patterns that differed in time in common ways, most probably driven by the individual effect of warming, while OA exhibited the most different responses throughout the experiment. Although the bacterial community at the acidified treatment OA exhibited various significant shifts in dominant OTUs over the 10–day experiment, shifts at the warm treatments W and GH were more consistent throughout the experimental period, showing a concrete impact of warming on community dynamics. Indeed, increased temperature was the manipulation that changed even the total autotrophic bacterial abundance (increase in *Synechococcus* numbers, Figure 2). The Shannon diversity index ranged between 2.9 and 3.7, with the lowest values reported at T0 and T3 at all mesocosms and the minimum at OA at T3 (Table 1), resembling the microcosm study by Aguayo et al. [59] that presented a low diversity index for the lowest–pH tanks. The impact of the experimental time was obvious from an ecological point of view, given that the >0.2 μm bacterial community patterns at the genus level were also driven by the concentrations of the inorganic nitrogen forms NO_3_– and NH_4_^+^ (distLM, adjusted R^2^ = 0.23, *p* < 0.05, Figure 3); the variations in nutrient concentrations were strongly related to the experimental time, since the ongoing biological processes within the mesocosm system (i.e., consumption and recycling via grazing and lysis) determine the quality and quantity of bio-available nutrients that, in turn, determine the synthesis of the communities too. In agreement with our results, during similar mesocosm experiments in the Mediterranean [60] and elsewhere [30,58], it was seen that the progression of time shaped the bacterial community composition in direct relation to phytoplankton-related alterations in organic matter and nutrient levels.

### 3.1. Immediate Treatment Effects at the >0.2 μm Bacterial Fraction (between T–1 and T0)

In terms of abundances, a unique instant effect of warming was visible at *Synechococcus*, as determined by flow cytometry; *Synechococcus* abundance significantly differed at W and GH (RM ANOVA, *p* < 0.05) and in specific, it was higher at W and GH compared to other mesocosms (Tukey HSD, *p* < 0.05) (Figure 2). Thus, the temperature benefited *Synechococcus* growth, which has also been reported elsewhere [7,25] including *Synechococcus spp.* cultural isolates from the coastal zone [26]. In our case, the positive effect was noticed early in the incubation and specifically up to T6. In a study by Bach et al. [34], a negative correlation between Cyanobacteria and pCO_2_ was seen early in the experiment when low-nutrient conditions were prevailing. In our case, *Synechococcus* abundance at T–1 averaged 2 × 10^4^ ± 1 × 10^3^ mL^−1^ and significantly increased up to 3 × 10^4^ ± 3 × 10^3^ mL^−1^ at T6 at W and GH (Figure 2). Competition between *Synechococcus* and other microbial cells and preferential grazing probably led to the loss of the positive effect on *Synechococcus* at T10. Indeed, it is often seen in the enclosed systems of mesocosms that indirect effects in the microbial dynamics mask the original effects of the treatments [11,30].

Between T–1 and T0, the pH was already decreased by 0.12 units, and the individual effect of the acidification was seen in the >0.2 μm bacterial community structure: Actinobacteria decreased in relative abundance at OA and GH (Figure 1), mainly due to the orders *Acidimicrobiales* and *Actinomycetales*. A recently described strong relation between pH and Actinobacterial strains [61] may be the reason for this sharp change in their relative abundance. At the same time interval (T–1 to T0), Bacteroidetes decreased down to half of its initial relative abundance in all mesocosms (Figure 1), reinforcing the importance of trophic status when interpreting the impacts of acidification; in fact, very contrasting results have been published so far for Bacteroidetes. On the one hand, under nutrient-rich conditions, Roy et al. [55] reported enhanced presence of Bacteroidetes upon increased pCO_2_ and Lindh et al. [27] reported variable responses upon increased pCO_2_ and temperature. On the other hand, Lin et al. [58] and our study performed under oligotrophic conditions report a decrease in Bacteroidetes at the acidified mesocosms (OA and GH). The driver taxa for this decrease were the order *Flavobacteriales* (*Flavobacterium* genus) and other genera of the *Flavobacteriaceae* family as well as the orders *Bacteroidales* and *Sphingobacteriales* (*NS11-12* family). Interestingly, Bacteroidetes recovered only at OA at T10, indicating that special organic matter released in the acidified mesocosms possibly benefited its growth at that time (discussed also below, see 3.2. chapter). The importance of studying the instant vs. longer-term effects of acidification was particularly highlighted by the evolution of Flavobacteria, with its relative abundance exhibiting different patterns at early vs. late stages of the experiment; at first it was decreased and later it recovered, resembling the late positive response of Flavobacteria at another mesocosm experiment in the Mediterranean [60]. Since Flavobacteria respond rapidly to phytoplankton fluctuations and play an important role in organic matter degradation [58] similar to Actinobacteria that play pivotal role in nutrient remineralization and recycling processes [61], their responses to pH manipulations require further investigation.

In expense to the above–mentioned decreased relative contribution of Bacteroidetes and Actinobacteria, Proteobacteria relative abundance gradually increased not only at OA and GH but also at C and W (Figure 1). Indeed, at T–1 the >0.2 μm fraction was half-dominated by Proteobacteria (53 ± 4%), followed by Cyanobacteria (26 ± 3%) and Bacteroidetes (15 ± 2%, Figure 1). Already at T0, Proteobacteria was positively affected by the incubation (61 ± 15% considering all mesocosms) and, especially, by the combined effect of temperature and pH manipulation (78% considering GH). Within Proteobacteria, the relative abundance of *Beta-* and *Deltaproteobacteria* remained nearly constant throughout the experiment (Supplementary Material, Figure S1) in contrast to Lindh et al. [27] that noticed a large effect on *Betaproteobacteria* that in our case constituted always <1% of the total 16S reads and was not affected by any treatment. As with Bacteroidetes, this difference could be related to the different nutrient levels between our experiments. It is noteworthy that the importance of trophic status was confirmed before within the oligotrophic-mesotrophic conditional range of the Mediterranean [17,22], as well as even within the seasonal nutrient gradients of the basin [21].

The combined effect of acidification and warming (GH) had a strong positive effect on total Proteobacteria but was particularly noticeable at the *Pelagibacteraceae* family (*SAR11* clade). *SAR11* started to increase immediately after the simultaneous pH and temperature treatment and ultimately reached up to 83% of the total 16S reads (maximum contribution at GH at T10). It is known that *SAR11* is exceptionally abundant in surface waters [62], and its presence is related to the dissolved organic matter quality [63], as well as to the temperature and salinity levels [64]. In partial agreement with our findings, Hartmann et al. [65] recently showed that *SAR11* could face even a sharp decrease in pH down to approximately 7.8 units, while its abundance was positively affected by the warming. Here, *SAR11* dominated the warm mesocosms (W and GH) but over-dominated the warm and acidified mesocosms (GH only), with gradually–increasing percentages over time. Considering that *SAR11* prevails under oligotrophic conditions, it was reasonable that its relative abundance increased between T–1 and T10, and that it negatively resembled the patterns of dissolved nitrogen; both NO_3_– and NH_4_^+^ concentrations decreased between T–1 and T10 (Table 1), offering an advantageous position for the oligotrophic *SAR11* clade. At the same time, PO_4_^3–^ concentration exhibited initial average concentration of 3.39 ± 2.51 nM, it largely varied between T0 and T10 at C, W and OA, while it was never fully consumed at GH (Table 1). The incomplete consumption of phosphate at GH, possibly linked to the fast exhaustion of ammonium in this treatment, may show a state of nutrient-limited growth that could justify the high occurrence of *SAR11* and the concomitant, opposite low representation of opportunistic taxa such as Flavobacteria.

During the short interval between T–1 and T0, the initially–rare phylum GN02 (<0.5% of the 16S reads), previously uncharacterized and uncultivated, also known as Gracilibacteria with unusual and largely uncharacterized metabolic capabilities [66] was also enhanced by acidification and warming. Specifically, the class *BD1-5* increased up to 10 and 15% at C and W, respectively, while it moderately increased to 4% at OA, and it was not increased at GH (Figure 1). Since the time scale of pH change is not realistic in most of the published experimental designs and here too (too rapid), these results should not be used to make overwhelming inferences.

### 3.2. Short-Term Treatment Effects at the >0.2 μm Bacterial Fraction (between T3 and T10)

In the middle of the experiment (T3), the pH drop already well-stabilized to 0.25 units compared to the initial levels; at this time, the bacterial community composition did not differ between the treatments and mild differences were seen at the following days (T6 and T10). In most cases, these differences did not reflect the community alterations of the first days, and occasionally, they were opposite. Altogether, the mesocosms shared several similarities at T6 and T10: the bacterial community seemed to be more able to adjust to sole temperature manipulation, reaching a new state at T10 that closely resembled the initial one, while it was less resilient to sole pH manipulation. In contrast to the other treatments, at OA the representation of unique OTUs was noticeable at T10. Despite the differences, the community did not seem to collapse, and the abundance of total bacterial cells remained in the same levels at all treatments. Thus, a stable community under acidified conditions—different from the original one—seemed to have been formed in the short experimental time scale.

In more details, a noticeable negative effect of the acidification was seen for the total *Alphaproteobacteria* relative abundance, which was lower at OA compared to the other mesocosms at T6 and T10 (Supplementary Material, Figure S1). This was not the case for all members of the class; on the one hand, the *SAR11* clade exhibited a large decrease at OA but on the other hand, several families remained constant, while the *Rhodobacteraceae* and *Hyphomonadaceae* families and the *Kiloniellales* order were enhanced at OA. Lin et al. [58] reported the same negative effect on *Rhodobacteraceae* upon acidification even under much more elevated nutrient concentrations compared to our system, while Aguayo et al. [59] did not notice an effect on this family at either a lower or higher pH manipulated-level in a microcosm study. It is remarkable that different pH levels have previously led to different responses even between phylogenetically closely–related *Rhodobacterales* taxa [58]. Consequently, it is not surprising that in our study the effect of the acidification was noticeable but was not homogeneous for phylogenetically related taxa.

*Gammaproteobacteria* increased in relative abundance at OA at T6 (orders *Alteromonadales* and *Oceanospirillales*) and T10 (orders *Alteromonadales*, especially *OM60* and *Chromatiales*), similar to an under-ice Arctic experiment [67] but not a mesocosm study in the same region [57]. Further pointing out the acidification impact, we noticed that at T10 the pH differences between C-W and OA-GH were still similar to the targeted one (0.21 units, Table 1) and then, three phyla with generally low relative contributions (Bacteroidetes, Cyanobacteria and Planctomycetes) retained a significantly higher percentage at OA (7, 6 and 1%, respectively) compared to the other treatments (1± 0.1%, 3 ± 1% and 0.4 ± 0.4%, respectively, considering C, W and GH). As discussed above, the phylum Bacteroidetes recovered to half of its initial percentage contribution only at OA at T10, which is most probably related to indirect phytoplankton community derivatives. The total chlorophyll concentration was at similar levels between GH and OA at T10 (Table 1) and was significantly higher compared to C and W (one–way ANOVA, post hoc Tukey test, p<0.05). Thus, a more detailed view of the phytoplankton community dynamics in the mesocosms may help us to relate specific bacterial strains with phytoplankton organic matter production (P. Pitta, in preparation). A hint towards this direction is presented in the work of D’ Amario et al. [39] that demonstrates particular impacts on coccolithophore calcification in the OA treatment.

Planctomycetes was dominated by the uncharacterized order *agg27*, while Bacteroidetes by the *Flavobacteriaceae* and *Saprospiraceae* families. Other studies did not relate the increase in *Flavobacteria* to the increase in pCO_2_ [27,55,57,58], since the increase in this taxa occurred upon nutrient addition or phytoplankton bloom. In any case, *Flavobacteria* played a key role in the acidified mesocosms by sustaining high connectivity within the community [57] and as explained above, this was possibly the case in the current experiment too.

Effects were seen upon experimental warming; the “other” phyla reached up to 1.2% relative abundance at T10 at W, mainly due to the presence of Firmicutes, Chloroflexi and ZB3.

Despite these taxonomic differences, the abundances of heterotrophic bacteria did not significantly differ between the controls and treatments. In agreement with other mesocosm experiments where the pCO_2_ was manipulated to reduce the pH in the Mediterranean [17,18] and elsewhere [11,15], the heterotrophic bacterial community exhibited diversity alterations that did not affect the total abundances. However, the functional potential of the total community was affected, as confirmed by the transcriptomics analysis (D. Miller, in preparation) and estimation of enzymatic activity and bacterial production (P. Pitta, in preparation) performed in the current study and as seen in other studies [33]. The enzymatic activity and bacterial production were also altered in previous experiments in the oligotrophic Mediterranean [17,40]. At T–1, the heterotrophic bacterial abundance averaged 5 × 10^5^ ± 1 × 10^4^ mL^−1^, it remained constant until T0 and experienced a common decrease until T3 and a common increase until T10 in all mesocosms (Figure 2).

### 3.3. Short-Term Treatment Effects at the 0.1–0.2 μm Bacterial Fraction (between T3 and T10)

This was the first time that <0.2 μm bacteria were tested for differences upon temperature and acidification manipulation. Bacteria smaller than 0.2 μm are supposed to be cells either actually smaller in size or dormant/starvation forms that may become larger upon nutrient enrichment [68]. In both cases, it is important to know the response of bacteria upon an experimental rise in the temperature and pCO_2_, since the smallest planktonic members seem to play a vital role in oligotrophic waters balancing carbon and biomass production and export [37]. Interestingly, the 0.1–0.2 μm fraction was over-dominated by Proteobacteria (98 ± 1% considering all samples), and the community patterns did not differ between treatments at T3, T6 and T10 (Figure 4). Within Proteobacteria, *SAR11* was dominant (77 ± 20%) and then, a high appearance of *Burkholderiaceae* (6 ± 11%), *Comamonadaceae* (2 ± 4%) and *Halomonadaceae* (5 ± 4%) was also observed. A low relative abundance of *SAR11* was seen at T3 at C and W (56 and 74%, respectively) when *Burkholderiacea* and *Comamonadacea* contributed more. In general, *SAR11* at the 0.1–0.2 μm fraction resembled the evolution at the >0.2 μm fraction too (i.e., gradual increase with time in the warm mesocosms) apart from a surprisingly low relative abundance at T10 at GH (47%). While *SAR11* was so low at T10 at GH (0.1–0.2 μm fraction), other taxa seemed to benefit from the pool of phosphate and chlorophyll, i.e., the families *Caulobacteraceae* (up to 5%), *Bradyrhizobiaceae* (up to 5%), *Halomonadacea* (up to 6%), *Rhodospirillaceae* (up to 1.5%) and *Oxalobacteraceae* (up to 1.5%), while the grant majority was attributed to *Burkholderiaceae* (up to 44%) and *Comamonadaceae* (up to 12%).

## 4. Conclusions

Climate change, including the rise in sea-surface temperatures and the drop in sea-surface pH, is expected to have drastic consequences on ecosystem functioning, especially in oligotrophic regions. During the current mesocosm experiment, we studied the individual as well as the synergistic effects of ocean warming and acidification at the bacterial community composition at the genus level at two fractions (0.1–0.2 and >0.2 μm). Our aim was to describe the synthesis of bacterial community in order to picture the functioning of the environment under the prospecting climate change scenarios. We found that the tiniest bacteria did not significantly change in their overall composition under any treatment, although a few taxa were slightly enhanced in the “greenhouse” mesocosms after 10 experimental days. In contrast, the >0.2 μm bacterial community did change with treatment and time. Several taxonomic differences were seen immediately after pH and temperature manipulation, but this effect was not maintained until the end of the experiment. In specific, as fast as one day after manipulation, Actinobacteria and Bacteroidetes were inhibited upon the decrease in pH, while the *SAR11* clade largely benefited from the combined decrease in pH and increase in temperature, possibly related to the fast exhaustion of nitrogen sources and an oligotrophic turn in the “greenhouse” mesocosms. Three days after manipulation, the bacterial community seemed to reach an equilibrium state, since nearly the same taxa prevailed at all mesocosms. Later on, the equilibrium state collapsed, revealing different-from-the-initial effects of acidification on Bacteroidetes, Cyanobacteria and Planctomycetes (enhancement) and *SAR11* (inhibition) as well as a large variability within *Gamma*- and *Alphaproteobacteria*. In fact, it was remarkable that even within the short 10-day experimental time period, there were fluctuations in the relative abundance of individual OTUs directly related to the trophic status of the incubated seawater and particularly to the levels of dissolved nitrate and ammonium. The strong dependence on nitrogen forms confirmed the importance of the abiotic parameters when interpreting microbial responses to pH and temperature manipulations.

The singular manipulation of pH seemed to have the most drastic effect on bacteria until the end of the experiment, enabling new taxa to take the foreground. Although we do not have information concerning the metabolic activity of these taxa, their differential functions in the food web (i.e., nutrient uptake and remineralization, susceptibility to lysis and grazing) can probably reason the different C-to-N ratios of exported material that is already measured in response to acidification [14]. These effects on heterotrophic bacteria were only seen at the OTU level: in terms of numbers, the heterotrophic bacteria did not change, and it was only the abundance of *Synechococcus* that increased upon warming. Similar to most of the taxonomic differences, the effect on *Synechococcus* vanished 10 days after the treatment. Altogether, this study highlights that bacteria exhibit community alterations upon sharp changes in pH and temperature levels; acidification itself sustained an impact on bacterial communities, a result that confirms that major oceanic biogeochemical processes will be affected by climate–related changes. Temperature and pH changes will occur at a slower rate than the one tested here, and so the current experimental design and study does not enable for an assessment of an adaptation potential, or for a clear reflection of climate change impacts. This study provides the floor for longer-duration studies, where CO_2_ levels will gradually increase, offering the opportunity to consider the oligotrophic open–ocean and the yearly–based fluctuations in phytoplankton community and nutrient levels.

## Figures and Tables

**Table 1:** Temperature (°C), pCO_2_ (μatm), pH, the concentrations of the inorganic nutrients NO_3_^–^ (μΜ), NH_4_^+^ (μM), PO_4_^3^– (nM),chlorophyll a (Chl, μg L^−1^) and the Shannon diversity index in the mesocosms. The pCO_2_ concentration was not determined (nd) at T–1 for C1 and W1. The Shannon diversity index was not determined (nd) for samples T6 OA2 and T10 OA2 due to the low sequence reads. C: control mesocosms, W: mesocosms where temperature was increased by 3 °C (named after warming), OA: mesocosms where pH was decreased by 0.3 units (named after ocean acidification), GH: mesocosms where temperature was increased by 3 °C and pH was decreased by 0.3 units (named after greenhouse).

Mesocosm	Temperature	pCO_2_	pH	NO_3_–	NH_4_^+^	PO_4_^3–^	Chl	Shannon Index
T–1 C1	25.10	nd	8.11	0.13	0.10	1.9	0.04	3.5
T–1 C2	25.10	369	8.11	0.12	0.05	1.6	0.04	3.6
T–1 W1	27.06	nd	8.11	0.06	0.28	3.1	0.05	3.5
T–1 W2	27.06	364	8.11	0.04	0.14	4.1	0.04	3.5
T–1 OA1	25.12	371	8.11	0.05	0.04	1.1	0.03	3.8
T–1 OA2	25.12	366	8.11	0.08	0.08	1.6	0.04	3.6
T–1 GH1	27.04	362	8.11	0.08	0.16	7.5	0.05	3.5
T–1 GH2	27.04	371	8.10	0.48	0.15	6.2	0.05	3.6
T0 C1	25.03	418	8.07	0.13	0.02	5.0	0.05	3.6
T0 C2	25.03	403	8.08	0.14	0.03	9.9	0.06	3.6
T0 W1	27.46	425	8.06	0.14	0.04	4.6	0.05	3.3
T0 W2	27.40	420	8.06	0.11	0.02	5.3	0.05	3.4
T0 OA1	25.02	549	7.97	0.03	0.011	3.9	0.06	3.4
T0 OA2	25.02	533	7.98	0.12	0.06	3.8	0.06	3.5
T0 GH1	27.35	612	7.93	0.07	0.04	4.9	0.06	3.5
T0 GH2	27.34	611	7.93	0.18	0.05	7.3	0.06	3.4
T3 C1	25.04	416	8.07	0.01	0.08	1.8	0.07	3.6
T3 C2	25.01	420	8.07	0.13	0.06	3.1	0.07	3.3
T3 W1	28.27	397	8.08	0.10	0.03	4.1	0.07	3.4
T3 W2	28.25	411	8.07	0.03	0.04	3.5	0.07	3.3
T3 OA1	25.05	794	7.84	0.07	0.06	3.1	0.09	2.9
T3 OA2	25.02	768	7.85	0.06	0.04	3.0	0.10	3.1
T3 GH1	28.24	803	7.83	0.05	0.00	5.6	0.10	3.5
T3 GH2	28.25	872	7.80	0.06	0.00	5.5	0.10	3.5
T6 C1	25.16	451	8.04	0.02	0.03	2.6	0.04	3.4
T6 C2	25.09	447	8.04	0.02	0.03	1.8	0.05	3.5
T6 W1	28.41	442	8.05	0.06	0.01	2.6	0.04	3.6
T6 W2	28.32	476	8.02	0.06	0.00	2.1	0.04	3.7
T6 OA1	25.27	805	7.83	0.02	0.01	3.0	0.05	3.6
T6 OA2	25.02	816	7.83	0.03	0.02	1.9	0.05	nd
T6 GH1	28.26	874	7.80	0.05	0.00	1.6	0.04	3.9
T6 GH2	28.41	887	7.80	0.04	0.00	2.4	0.04	3.5
T10 C1	25.26	438	8.05	0.02	0.00	4.4	0.04	3.8
T10 C2	25.23	442	8.05	0.06	0.00	2.6	0.05	3.4
T10 W1	27.86	437	8.05	0.05	0.00	3.6	0.05	3.3
T10 W2	27.92	456	8.04	0.00	0.00	3.9	0.04	3.8
T10 OA1	25.29	785	7.84	0.02	0.00	3.4	0.05	3.7
T10 OA2	25.22	793	7.84	0.03	0.00	2.6	0.06	nd
T10 GH1	27.90	812	7.83	0.03	0.00	5.5	0.06	3.5
T10 GH2	27.96	816	7.83	0.05	0.00	4.4	0.07	3.2

## Data Availability

The raw sequences have been submitted to the European Nucleotide Archive (ENA) database with accession number ERA19781743 (runs between ERR10742502 and ERR10742565). The datasets of the environmental parameters used in this work are available at https://doi.pangaea.de/10.1594/PANGAEA.836005.

## Supplementary Materials

The following supporting information can be downloaded at: https://www.mdpi.com/article/10.3390/microorganisms11020273/s1, Figure S1: Percentage contribution of Proteobacteria classes in the mesocosms at T–1, T0, T3, T6 and T10 at the >0.2 μm fraction (over the total Proteobacteria reads).
