# Supplementary material for: Responses of Free-Living Planktonic Bacterial Communities to Experimental Acidification and Warming
Source: Microorganisms. 2023 Jan 20;11(2):273. doi: 10.3390/microorganisms11020273 (PMC9963540; doi:10.3390/microorganisms11020273)
Supplement: Supplementary file 1 [file microorganisms-11-00273-s001.zip › microorganisms-2054793-supplementary.pdf]

APPENDIX

Supplementary Figures

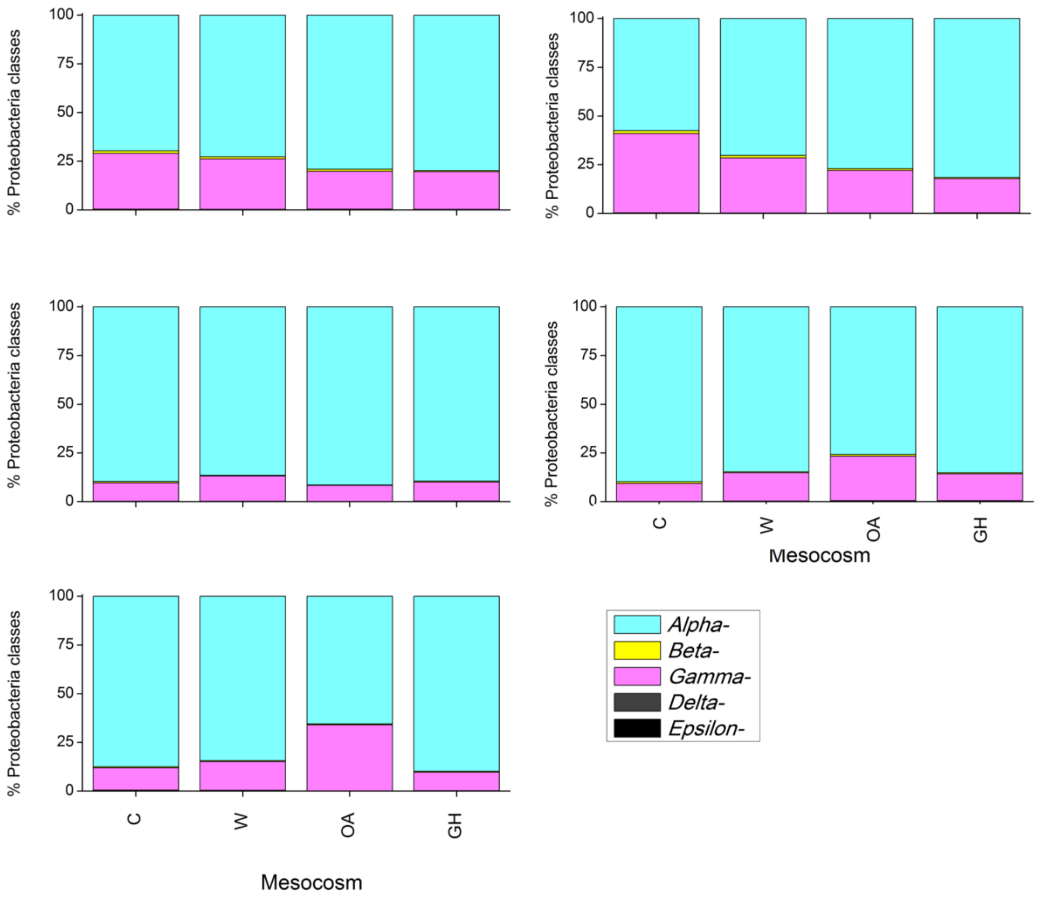

Supplementary Figure S1. Percentage contribution of Proteobacteria classes in the mesocosms at T-1, T0, T3, T6 and T10 at the >0.2 μm fraction (over the total Proteobacteria reads). Delta- and *Epsilonproteobacteria* never exceeded 0.5 and 0.02% of the Proteobacterial reads, respectively. *C: control mesocosms, W: mesocosms where temperature was increased by 3 °C (named after warming), OA: mesocosms where pH was decreased by 0.3 units (named after ocean acidification), GH: mesocosms where temperature was increased by 3 °C and pH was decreased by 0.3 units (named after greenhouse).*
